# Patient and health practitioner views and experiences of a cancer trial before and during COVID-19: qualitative study

**DOI:** 10.1186/s13063-022-06453-z

**Published:** 2022-06-18

**Authors:** Frances C. Sherratt, Peter Fisher, Amy Mathieson, Mary G. Cherry, Andrew R. Pettitt, Bridget Young

**Affiliations:** 1 Department of Public Health, Policy & System, University of Liverpool, Liverpool, UK; 2 Department of Primary Care and Mental Health, University of Liverpool, Liverpool, UK; 3 NIHR Applied Research Collaboration (ARC) Greater Manchester, Centre for Primary Care and Health Services Research, University of Manchester, Manchester, UK; 4 Department of Molecular and Clinical Cancer Medicine, University of Liverpool, Liverpool, UK; 5 The Clatterbridge Cancer Centre NHS Foundation Trust, Liverpool, UK

**Keywords:** Equipoise, Uncertainty, COVID-19, Pandemic, Communication, Clinical trials, Randomised controlled trials, Haematology, Lymphoma, Life change events

## Abstract

**Background:**

Understanding patient and health practitioner perspectives on clinical trials can inform opportunities to enhance trial conduct and design, and therefore patient experience. Patients with haematological cancers have faced additional risk and uncertainty during the pandemic but it is unclear how they and practitioners have experienced cancer trials during this period. In the context of a haemato-oncology trial (PETReA), we compared patient and practitioner views and experiences of PETReA before and during COVID-19.

**Methods:**

Qualitative study embedded within PETReA. Semi-structured interviews (*N*=41) with patients and practitioners from 16 NHS sites before (*n*=17) and during the first wave of COVID-19 (*n*=24). Analysis drew on the framework approach.

**Results:**

Practitioners acknowledged the need for the trial to continue during the pandemic but their treatment preferences altered, becoming more pronounced for patients who had a favourable response to induction treatment, while staying unchanged for patients with a less favourable response. Practitioners commented that COVID-19 meant the evidence base for the trial arms was lacking or mixed, but that it likely increased the risks of maintenance treatment for patients with a favourable response to induction treatment. While only one participant interviewed withdrew from PETReA during the pandemic, others said they would consider withdrawing if information that they were at increased risk of severe illness from COVID-19 became available. During COVID-19, patients described less frequent contact with the trial team, which left some feeling less clear about their trial pathway. However, several described having in-depth, collaborative discussions with practitioners about the risks and benefits of randomisation in the context of COVID-19. Patients valued these discussions and were reassured by the emphasis practitioners placed on patients being free to withdraw if circumstances changed, and this helped patients feel comfortable about continuing in PETReA.

**Conclusions:**

The findings point to ways trial communication can support patients to feel comfortable about continuing in a trial during uncertain times, including adopting a more in-depth, collaborative exploration of the risks and benefits of trial arms with patients and emphasising voluntariness. The results are relevant to trialists recruiting patients who are clinically extremely vulnerable or are at increased risk of poor COVID-19 outcomes despite being vaccinated.

**Supplementary Information:**

The online version contains supplementary material available at 10.1186/s13063-022-06453-z.

## Introduction

There are many definitions of ‘patient experience’ but four key concepts are viewed as central [1, 2]: (a) the sum of all interactions (b) shaped by an organisation’s culture (c) which influences patient perceptions (d) across the continuum of care. Anhang Price et al. [3] used the term ‘patient experience’ to refer to any process observable by patients, including subjective experiences (e.g. perception of pain control), objective experiences (e.g. waiting times), and observations of health practitioner behaviour (e.g. clinical communication). Enhancing patient experiences and ensuring healthcare is patient-centred is intrinsically important [4]. It is also positively associated with clinical effectiveness and patient safety [5] and should therefore be considered a core element of quality healthcare.

In the context of clinical trials, qualitative studies have examined patient experiences to identify opportunities to enhance trial conduct, with the ultimate aim of improving informed consent and recruitment [6, 7]. These studies often analyse audio-recorded trial consultations, sometimes supplemented with patient and practitioner interviews, to identify and address challenges that practitioners encounter in communicating trials. What is said to patients and how it is said can vary considerably during trial consultations [8]. For example, previous research has explored how practitioners communicate about ‘clinical equipoise’ with patients. Clinical equipoise refers to genuine uncertainty about the relative clinical merits of trial arms, which is necessary to recruit patients to a trial [9]. Despite practitioner intentions to set aside personal biases and neutrally convey trial arms, equipoise is commonly omitted or compromised in trial discussions [10]. Imbalanced presentation of trial arms can influence patient views towards trial arms and willingness to participate in a trial [11].

On 11th March 2020, the exponential increase in COVID-19 cases worldwide caused by the novel severe acute respiratory syndrome coronavirus 2 (SARS-CoV-2) led to the World Health Organization declaring a pandemic [12]. This was followed by efforts in almost all countries to protect the public and reduce the spread of the virus, especially to ‘high-risk’ individuals who were expected to have worse outcomes. In the UK nations, selected groups of patients perceived to be clinically extremely vulnerable to adverse COVID-19 outcomes were advised to ‘shield’ (i.e. not leave the house except for medical appointments and socially distance from other household members) from 21 March until 31 July 2020 and again from 6 January until 31 March 2021 [13].

COVID-19 has presented unique challenges for the clinical trial community. Many trials were forced to pause recruitment to minimise the number of patients visiting hospitals and enable clinical staff working on trials to be redeployed to support frontline services [14]. Cancer trials were significantly affected; approximately 95% of Cancer Research UK trials were either paused completely or paused in some of their trial sites at the peak of the pandemic in 2020 [15]. COVID-19 presented cancer patients with added risk and uncertainty. Overall, patients with cancer are at increased risk of COVID-19 infection and associated serious complications [16], whilst patients with haematological cancers (e.g. follicular lymphoma) have additional risk of COVID-19 mortality, compared with all cancer patients [17–19]. Emerging evidence suggests that certain patient groups, such as those with haematological cancers [20] are far less likely to gain adequate protection from COVID-19 vaccines. Further to this, patients undergoing certain cancer treatments, such as anti-CD20 monoclonal treatment, are at increased risk of poor COVID-19 outcomes despite vaccination [21].

**Fig. 1:**
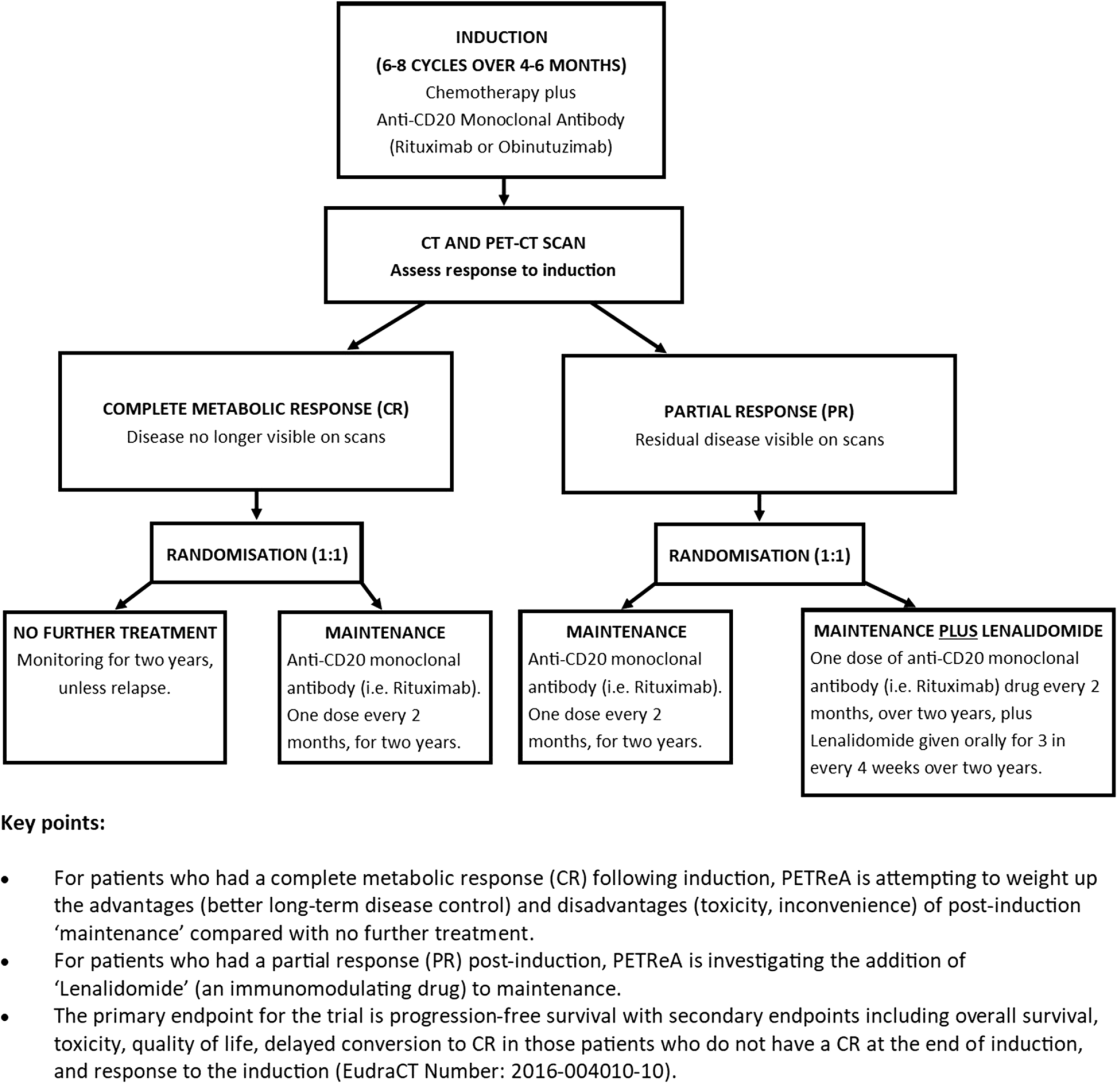
Overview of the PETReA trial

PETReA (Phase 3 evaluation of PET-guided, Response-Adapted therapy in patients with previously untreated, high tumour burden follicular lymphoma) is an ongoing, international, multicentre, non-blinded, phase III randomised controlled trial. It is testing whether PET (positron emission tomography) scans can help determine which patients need anti-CD20 monoclonal treatment (referred to as ‘maintenance’ from hereon) after first-line induction treatment (referred to as ‘induction’ from hereon) for follicular lymphoma. PETReA is registered on the EU Clinical Trials Register (2016-004010-0) and ISRCTN (ISRCTN86739591) and Fig. 1 and the glossary provide further details. Simultaneously with the advice on shielding during the first COVID-19 wave, guidance was published by the National Institute for Health and Care Excellence (NICE) [22] and NHS recommending changes to cancer management and treatment during COVID-19 surges to attempt to reduce viral exposure, minimise suppression of the immune system due to cancer treatment (i.e. iatrogenic immunosuppression), and increase capacity in secondary care to manage COVID-19 surges. The guidance was supplemented by a list of NHS England-approved interim treatment options, several of which related to haematological cancers [22]. In accordance with national guidance, recruitment into the PETReA trial was paused for 3 months from the start of the first COVID-19 wave (the end of March 2020 onwards), and the protocol amended in several ways to reduce potential risks to patients.

Practitioners’ personal views about trial arms can impede their ability to convey equipoise [23, 24], and it is unclear whether and to what degree COVID-19 may have influenced this ability when recruiting clinically extremely vulnerable patients to a trial. The emergency phase of the pandemic may have receded in countries with high rates of population immunity. Nevertheless, given the additional risk and uncertainty that patients with haematological cancers have faced during the pandemic and evidence that the vaccine offers such patients weaker protection, it remains important to examine patient views and experiences of trials. Such evidence can help improve trial conduct and recruitment for vulnerable patients in the ongoing context of COVID-19. Additionally, comparing patient experiences of taking part in PETReA before and during COVID-19 may have wider lessons for improving communication about clinical trials. In the context of PETReA, we aimed to (1) examine and compare practitioner views and experiences of randomising patients before and during COVID-19 and (2) examine and compare patient views and experiences of participating in a haemato-oncology trial before and during COVID-19.

## Methods

### Study design and setting

We adopted a qualitative approach, conducting and analysing semi-structured interviews with patients and practitioners to provide detailed insights on their views and experiences [25] of PETReA. Semi-structured interviews are an established qualitative methodology suitable for investigating the views and experiences of participants [26], and allowing interviewers to explore key issues of relevance to the study research questions, whilst enabling participants to raise their own pertinent issues that the interviewers may not have anticipated. A National Research Ethics Committee (17/NW/0512) approved PETReA, including the embedded qualitative study.

PETReA started recruitment in May 2018. As of 5th January 2022, recruitment is ongoing in 50 sites across the UK and Australia and 321 patients had been recruited of the 1000 targets. The qualitative study was conducted at 30 sites in the UK. These were selected into the qualitative study based upon the order in which sites opened to PETReA. The qualitative study ran from April 2018 to October 2020. Patients who were eligible for PETReA were also eligible for the qualitative study; they could take part in PETReA, the qualitative study, both or neither.

### Participants and procedure

#### Patient interviews

Patients were eligible to be interviewed for the qualitative study if they had been approached and were eligible to participate in PETReA. Practitioners (haematologists, research nurses, and clinical trials practitioners) initially invited patients to participate in PETReA shortly before the patient started induction and they also obtained written consent for the patient’s contact details to be shared with the qualitative study researchers (FCS and AM, who are both qualitative researchers with backgrounds in health research). We purposively sampled with the aim of interviewing a range of patients, including those who declined or consented PETReA, had a complete metabolic response (CR) or partial response (PR) to induction, and allocated to each of the patient pathways (i.e. no further treatment, maintenance, or maintenance plus Lenalidomide). We also included patients of different sex, age, hospital site, socio-economic status, although we did not record ethnicity.. FCS and AM subsequently contacted patients after they had been randomised or declined PETReA to provide them with a participant information sheet and invite them to participate in an interview.

Patients who participated in PETReA were interviewed within a few months of being randomised and patients who declined PETReA were invited within a few months of having the trial discussion. FCS and AM conducted and audio-recorded semi-structured patient interviews, either face-to-face (before COVID-19), by telephone, or video call, after first obtaining their consent. Interviews were topic guided but the guide was developed in the light of ongoing analysis. Table 1 lists the topics explored in the patient and practitioner interviews. Supplementary File 1 is an example topic guide used in the semi-structured interviews and indicates the type of the questions asked.

**Table 1:** Overview of the topics explored in the patient and practitioner interviews

**Patient interviews**	
Symptoms and diagnosis	
Initial approach about PETReA	
How the practitioner described PETReA	
Patient’s own treatment preferences and their perceptions of their practitioner’s treatment preferences	
Reasons for declining, taking part, and/or withdrawing	
Experience of having the initial treatment	
Views and experience of having a PET-CT scan	
How the practitioner described the results of the PET-CT scan	
How the practitioners described randomisation and treatment allocation	
Views and understanding about randomisation	
Views on their allocated treatment pathway	
Reflections on PETReA since being approached	
**Practitioner interviews**	
Clinical role and involvement in PETReA	
Initial views about PETReA and sources of information	
Patient pathways in and out of PETReA	
Experiences of approaching patients about PETReA	
Practitioner’s own treatment preferences and their perceptions of patient treatment preferences	
Views about PET-CT scanning	
How they described the results of the PET-CT scan	
How they described randomisation and treatment allocation	
Experiences of delivering treatments in and out of PETReA	
Comments on the qualitative study (e.g. how they would prefer to receive feedback)	

#### Practitioner interviews

Practitioners were eligible to be interviewed if they had approached a patient about PETReA. FCS identified eligible practitioners by checking investigator details on PETReA consent forms, liaising with trial coordinators, and contacting local principal investigators directly. We purposively sampled to include a range of practitioners, based on role (haematologist, research nurse, clinical trials practitioner), sex, and hospital site. FCS and AM approached practitioners by telephone or email to provide them with a participant information sheet and invite them to participate in an interview. FCS and AM followed the same procedure for conducting the practitioner interviews as they did with patients, except for using a topic guide specifically for practitioners. Again, an overview of the topics explored with practitioners can be found in Table 1.

#### Analysis

All audio-recorded interviews were transcribed, checked and pseudoanonymised. We continued to collect data until we achieved an adequate sample size, as guided by the principles of ‘information power’ [27]. Data analysis was interpretative and iterative, drawing on thematic analysis [28] and the framework approach [29]. A thematic approach to analysis was well-suited, as the analytic interest focused on identifying themes across the dataset on how participants’ experiences were located within the wider socio-cultural context [30]. Data analysis also drew on the constant comparison method [31], and the patients’ and practitioners’ accounts were analysed separately. FS led all aspects of the analysis, with BY, PF and MC contributing to the analysis to enhance analytical rigour [32]. FS listened to audio-recordings of interviews to consider subtleties, such as intonation, and familiarise herself with the data. FS read and re-read transcripts with BY, PF and MC each reading a subset of transcripts and meeting periodically to develop and refine the analysis. Through constant comparison across transcripts, FS coded the transcripts by hand to generate initial codes and used Microsoft Excel to organise the dataset, compare findings between sampling criteria, and assist with the analysis (e.g. searching for data corresponding to themes and reviewing themes) [33].

FS, BY, PF and MC were members of the PETReA Trial Management Group (TMG) and attended TMG meetings regularly, which were also attended by NHS site principal investigators and other health practitioners involved in trial recruitment. Akin to previous qualitative studies embedded in trials (e.g. [34, 35]), the qualitative team regularly presented the TMG with emerging findings to inform trial communication practice, with the aim of improving patient experiences of trial communication and recruitment.

Illustrative quotes are presented with identifiers, detailing whether a participant was a patient (P) or practitioner (HP), interviewed before or during the pandemic, and for patients only, whether they had a complete metabolic response (CR) or partial response (PR) to induction, and which pathway they were allocated to (i.e. no further treatment [None], maintenance, or maintenance plus Lenalidomide [Len]).

## Results

### Participant characteristics

FCS and AM attempted to contact 30 patients from 13 UK NHS sites for interview. Of those, 26 patients from 12 sites participated. Of the four who did not participate, one patient agreed to interview but did not attend and the other three did not respond to initial contact.

Table 2 provides an overview of participant characteristics. Approximately half of the patients were female (*n* = 14, 53%), and their median age was 62 years (range, 42–75). Of the CR participants (17/25, 68%), 9 were randomised to no further treatment and 8 were randomised to maintenance (2 of whom withdrew following randomisation). Of the PR participants (8/25, 32%), half were randomised to maintenance plus Lenalidomide and the other half were randomised to maintenance only.

**Table 2:** Participant and data characteristics

**Patient interviews**	***N*** **= 26**
Age	
Median years (range)	62 (42–75)
Sex	
Females/males	14/12
Index of multiple deprivation decile^a^	
Most deprived (1–3)	4
Average deprivation (4–7)	11
Least deprived (8–10)	8
Format of interview	
Telephone	26
Duration of interview	
Median minutes (range)	61 (40–84)
PETReA trial participation status	
Consent (v declined)	25 (v 1)
PET-CT response to induction and trial arm allocation^b^	*n* = 25
Complete metabolic response (CR)	
No further treatment	9
Maintenance	8
Partial response (PR)	
Maintenance	4
Maintenance plus Lenalidomide	4
No. of participating UK sites	12
**Practitioner interviews**	***N*** **= 15**
Practitioner’s role	
Haematologist oncologist	10
Research nurses/clinical trial practitioners^c^	5
Format of interview	
Telephone	11
Face-to-face	3
Video call	1
Duration of interview	
Median minutes (range)	62 (32–81)
No. of participating UK sites	12
**Total interviews**	**41**
No. of participating UK sites	16

^a^The Index of Multiple Deprivation (IMD) deciles were available only for those patients living in mainland England (*n*=23/26). IMD ranks every small area in England from 1 (most deprived area) to 32,844 (least deprived area). The deciles are derived from the ranks and we divided these into most deprived (1–3), average deprivation (4–7), and least deprived (8–10). ^b^Trial participants only. ^c^Clinical trials practitioner is a reasonably new role in the NHS, which entails trial recruitment, education, support and monitoring of the patient entering a clinical trial

FCS and AM attempted to contact 30 practitioners (haematologists, research nurses, and clinical trials practitioners) from 19 UK NHS sites who were eligible for interview. Of those, 15 practitioners from 12 sites were interviewed. Of the fifteen who did not participate, three responded and agreed but did finalise a time, and 12 did not respond. Table 1 summarises participant characteristics.

In total, 11/26 (42%) patients and 6/15 (40%) practitioner interviews were completed before the pandemic.

### Qualitative findings

#### Increased practitioner hesitancy to randomise patients during COVID-19

Both before and during the pandemic, health practitioners explained that it was standard practice in the UK to offer maintenance treatment to CR patients. Before the pandemic, many practitioners described the benefits and drawbacks of both maintenance treatment and no further treatment in a balanced way. However, a few were less balanced and discussed the drawbacks of maintenance in detail, hypothesised that it was unnecessary, and indicated that other practitioners were starting to avoid maintenance outside of the trial:

One of my concerns with [maintenance] as we’re using it more just routinely, so off trial, … [is] the increased risk of infections with it. So that would be a concern as well for those that are randomised to [maintenance]. It’s just I generally have a lower threshold these days for… stopping [maintenance] prematurely because of those issues. (HP6_Before)

During the pandemic, practitioners became more hesitant about maintenance for CR patients. They described feeling relieved when such patients were randomised to no further treatment arising from concerns that maintenance might increase patients’ risk of severe illness from COVID-19, and reported that patients were also nervous about attending hospital during the pandemic due to the risk of COVID-19 infection:

I’m quite relieved when I don’t have people – at the moment – randomised to maintenance, fundamentally because I think patients are nervous about coming up to hospital… (HP4_During)

Before COVID, … the concern [patients] have is, “Well if it’s standard of care, and we’re stopping treatment [i.e. no further treatment], what if it doesn’t work?” You know, it’s the opposite… Stopping treatments is perceived as, probably, a greater risk to them… Whereas now, I think, the people that are being randomised … some of them are hoping for no more treatment so they don’t have to come to hospital anymore (HP13_During)

Practitioners spoke about factors such as patient age and comorbidities as influencing their views on trial arm suitability during the pandemic. Although no patients reported choosing to withdraw from PETReA, one practitioner described withdrawing a CR patient who was randomised to maintenance. This practitioner felt they had a duty of care to intervene as the patient was at risk of severe illness from COVID-19 due to their age and comorbidities:

They were in their late 70s… they were overweight, and they had [a comorbidity] and they [had a CR]. I just felt, on the basis of those problems and their disease situation, that stopping maintenance was appropriate for them. That’s the only one I’ve actually had. I, sort of, dictated what I thought was best… I just thought, “If [they] get COVID and he gets it badly, then I won’t forgive myself. (HP4_COVID)

For PR patients the pandemic did not change practitioner preferences on maintenance. Before and during the pandemic, no practitioners doubted the need for maintenance for this group of patients, and none expressed explicit preferences for either maintenance or maintenance plus Lenalidomide. Many described the potential side effects of maintenance plus Lenalidomide and questioned how well it might be tolerated in patients, but they also emphasised its use in treating similar patient populations and the potential benefits of maintenance for PR patients.

In addition to becoming more hesitant about randomisation to maintenance for CR patients during the pandemic, practitioners also described advising or offering patients the opportunity to stop induction early and progress to randomisation. In the context of COVID-19, they felt that patients may fare better if they completed only 4–5 cycles of induction followed by maintenance, compared with the standard 6 cycles. Speaking about a patient who was subsequently found to have a PR to induction, one practitioner described having stopped the patient’s induction early in response to the pandemic. They viewed maintenance plus Lenalidomide as “potentially less toxic than [induction], so risks of [COVID-19] infection are less” than induction and elaborated on the rationale for stopping induction early:

One of my patients… had four cycles… and then COVID hit. So I said, “You have had a good response to treatment, I do not think you should have any further [induction cycles], especially bendamustine [component of induction].” He is now on the [PR] [maintenance plus Lenalidomide] arm… I do not know, if he had a further two cycles of chemotherapy would he have been on the other arm, [CR to induction]? … Potentially [maintenance plus Lenalidomide] is less toxic than [induction], so his risks of infection are less. So, I think it is the right thing for him. (HP2_During)

#### Patient treatment preferences before and during COVID-19

Before and during the pandemic, although CR patients described being willing to be randomised, many welcomed the end of induction, expressed concerns about the potential side effects of maintenance (e.g. risk of infection), and preferred no further treatment. For example, a patient randomised before COVID-19 explained:

I'd considered… the long term effects (of induction) anyway of the fatigue and everything and decided, look, I don't want another two more years of this. Which I mean, inevitably the [maintenance] does have side effects… (PT26_Before_CR_Maintenance)

Expressing similar views, a patient randomised during COVID-19 commented:

I probably felt quite happy - I mean, about not getting any more treatment just because, you know, in spite of how smooth [induction] all was, yes, it becomes slightly tiresome when people have to keep sticking needles in you. So the thought of that not having to happen is fine. (PT6_During_CR_None)

Some CR patients did not understand the rationale for maintenance and thought it unnecessary for patients like them:

I think to myself, “If you’ve got a headache and you’ve taken your paracetamol, or ibuprofen or whatever, and your headache has got better, and you’re not in any pain, why would you want to carry on taking paracetamol?” … why would you want to carry on taking tablets when you don’t need it? (PT18_Before_CR_None)

In line with practitioners, CR patients who were randomised during the pandemic expressed additional pandemic-related concerns, and their preferences for no further treatment seemed to become more pronounced. Patients worried that maintenance might weaken their immune systems and increase their risk of severe illness from COVID-19:

[I said] Look, it is quite simple really. If I am randomised for treatment, I will withdraw from the trial because I don’t want to put myself in that situation. You know, further treatment will just further weaken me and, actually, I need to get strong. (PT1_During_CR_None)

They also focused on how maintenance would require them to shield to avoid getting COVID-19, which would impact on their quality of life:

It is all this quality of life stuff, isn’t it? You know, and if you feel you are going to be another two years in a very vulnerable place… we like to travel and do things like that. And, obviously, [being randomised to maintenance] would have been more limiting for me (PT13_During_CR_None)

In contrast, before the pandemic PR patients tended to focus on the benefits of the treatment to which they had been allocated or the drawbacks of the non-allocated treatment, and did not have a clear preference for either treatment arm. For example, a PR participant who was randomised to maintenance plus Lenalidomide described himself as “*hopeful*” that he would benefit from treatment (PT20_Before_PR_Len).

While the preferences of CR patients for no further treatment became more pronounced during the pandemic, the preferences of PR patients seemed unchanged:

I thought at the time, “Ah, okay, if I don’t have complete remission (CR), it would be interesting if I was randomised to have a go with these tablets [Lenalidomide] as well.”… I was very pleased actually that I’ve been chosen to have [Lenalidomide] as well. (PT4_During_PR_Len)

The types of concerns that PR patients expressed, such as vulnerability to severe illness from COVID-19 and having to shield, were similar to CR patients. However, PR patients were more accepting of the trial arm to which they had been allocated to compared with CR patients. PR patients focused on the risk of their disease progressing if they did not have treatment and how this outweighed the risk of developing severe illness from COVID-19:

The risk of me catching COVID is significantly less than me getting very, very ill from not having treatment (PT4_During_PR_Len)

[When randomised, I asked] Does that mean I’m going to have to shield for two years? … If lockdown becomes necessary again, then yes, I’m going to be extremely vulnerable, but I would have been anyway [irrespective of trial arm] (PT5_During_PR_Len)

#### Collaborative discussions about risk and emphasis on the opportunity to withdraw

When interviewed during the pandemic, several practitioners commented or alluded to the absence of clear evidence about how COVID-19 influenced the risks of induction and the trial arms, and emphasised the challenges this created in discussing and deciding treatment pathways with patients:

We are making decisions and thinking theoretically about what may or may not increase people’s risk [of COVID-19], based on very little evidence at all (HP4_During)

Despite this, neither practitioners nor patients spoke about the lack of evidence regarding treatments in the context of COVID-19 being made explicit in patient-practitioner discussions about the trial. One practitioner explained that although the uncertainties raised by the pandemic influenced discussions about the trial, their discussions with patients about PETReA during the pandemic were broadly similar to the discussions they had before the pandemic:

[Practitioner] was explaining it in the context of COVID, that at the moment the study is running, it might be that [the study] gets put on pause, and if you are on it you can carry on but the decision will be flexible, in line with what the pandemic is doing… [the pandemic] is always mentioned, but I would not say it has changed [consultations] as dramatically as you would have thought. (HP5_During)

As discussed above, practitioners often talked with patients about truncating induction cycles during the pandemic and progressing to randomisation as a way of mitigating COVID-19 related risks, and some described strongly recommending this to patients as the best option for them. Across sites, while some patients did not mention having received such advice, others commented that their practitioners had advised stopping induction early. Speaking of accepting his practitioner’s recommendation to stop induction before the final cycle, a patient commented:

After having the seventh cycle, I was now meant to do the eighth cycle when this COVID-19 campaign broke out. So [practitioner] advised that I would not receive that treatment anymore, because of the vulnerability to me with the COVID-19, and also looking at the blood sample… So, [practitioner] just [decided] not to give me that because of the situation. So I said, “Fine.” (PT7_During_PR_Maintenance)

Before and during the pandemic, most practitioners described the importance of emphasising to patients that they were free to withdraw from the trial. However, some indicated that they gave greater prominence to this in the context of the pandemic. Before the pandemic, few patients talked about their right to withdraw from the trial having been emphasised by practitioners. In contrast, during the pandemic, and in alignment with practitioner accounts, patients often focussed on how practitioners had emphasised they could withdraw from the trial at any time. Several patients noted that in the context of the pandemic this helped them to feel more confident about their decision to participate. A patient who had been worried about continuing with the trial at the start of the pandemic spoke of valuing the opportunity to discuss the trial and treatment with her practitioner and was reassured by the practitioner’s reminders that she could withdraw at any time:

Because the hospital was so dire [at the start of the pandemic], (Laughter)… There were beds all up around corridors. It was just awful… And then I had a chat with [research nurse] about it and she just said, “You don’t have to. You can step out any time you like.” But we had a good discussion and it was nice because I wasn’t just somebody on a trial. (PT1_During_CR_None)

The patient added that if she had been randomised to her least preferred arm (maintenance), further conversation and reassurance would have been essential to support her in continuing with the trial:

At (the start of the pandemic), I kind of like was more concerned, to be honest, about how the hospitals were coping with COVID and knowing how close the nurses have to get to you, and knowing the problems with PPE… there would need to be a lot more kind of conversation, I suppose, and reassurance before I could have gone ahead with that. (PT1_During_CR_None)

#### Being a participant on a haemato-oncology trial during COVID-19

Patients varied in terms of how susceptible to COVID-19 they perceived themselves to be, depending on factors, such as the nature of their work, where they lived, who they lived with, and their behavioural strategies to avoid COVID-19 transmission:

I know that the impact, probably, on me would be pretty severe if I did get COVID but, equally, especially at the moment living where I do… in the leafy suburbs outside (a city). If I'm reasonably well behaved, the chances of getting it are pretty minuscule, actually. (PT6_During_CR_None)

In line with practitioner reports, patients described feeling worried about catching COVID-19 during hospital visits, especially at the start of the pandemic when infection control measures were not always in place:

I went in for my last set of chemo at the height of the pandemic in [place]. It was just bizarre, so you can imagine how paranoid I was. If it weren’t for a packet of Valium I’d have been all over the place. (PT9_During_CR_Maintenance)

Later, the pandemic led to many consultations being undertaken by video call or phone rather than in person. Several patients felt that this influenced the quality of their consultations with the clinical team, although a few said they would have preferred a video consultation, compared to telephone consultation. Patients randomised to maintenance during the pandemic were less clear about what it would entail compared with patients before the pandemic and expressed uncertainties about when maintenance would start and how often they would receive treatment. Similarly, patients mentioned that they were waiting to hear from their clinical team about what the maintenance would entail:

[The clinical team will] probably have a better idea on exactly how the situation is standing with the hospitals and any dangers or any possibilities of catching [COVID-19] [during maintenance]. So I’ll be guided by what [practitioner] says (PT10_During_CR_Maintenance)

Furthermore, patients referred to the potential for the pandemic to change or delay their access to treatment:

Obviously they say after [the last induction cycle] it’s three months’ recovery and then they’ll probably call me back in, or they might do… depending on this COVID-19 (PT9_During_CR_Maintenance)

A patient who acknowledged some uncertainty about maintenance during the pandemic attributed this in part to her research nurse, who she viewed as a valuable source of information, no longer being available after the nurse was redeployed to COVID-19 clinical duties.

I think a lot of my slight feelings of uncertainty now (approaching maintenance) wouldn’t have been there if - through the last phases of my chemo, I would still have been seeing (research nurse) and I could have talked through things as they came along… a lot of this is just the result of coronavirus. (PT14_During_CR_Maintenance)

Although patients described new challenges in accessing treatment and care during the pandemic, they also felt relieved and grateful for the haemato-oncology clinical advice and treatment that they still had access to via PETReA during this time. Referring to the restrictions encountered by patients who required other services, a patient who chose to continue with her induction described how she was grateful for the opportunity to decide to continue accessing treatment:

[Practitioner] phoned me to say, “Look, [your lymph nodes] have gone down… you are going the right way… Do you want to carry on? In view of COVID, do you want to carry on with the treatment because I have to ask you, because, obviously, the risk of you coming into the [NHS site] and COVID, etc.,” and I said, “Undoubtedly, yes.” … [Practitioner] said, “No, that’s absolutely fine. Let’s carry on,” and we did… I’m very, very grateful actually that I’ve been able to carry on, because I know a lot of people with their treatments have stopped. (PT4_During_PR_Len)

Patients had been asked to shield and a few noted that this had impeded their physical recovery following induction, as it restricted opportunities to exercise. However, one patient who had been encouraged to shield during treatment irrespective of the pandemic, described the way shielding became a shared experience as the country went into lockdown, which she found comforting:

I know during chemo, I couldn’t go out during certain times because my immunity was so low. So, I was, sort of, going through a lockdown of my own, but I wasn’t expecting the rest of the world to come through lockdown with me… it was really helpful because people were in shock and the news about the pandemic, and I was actually going through this anyway, regardless of what was happening outside there. (PT4_During_PR_Len)

#### Running a cancer trial during COVID-19

Practitioners indicated that their worries about patients’ COVID-19-related risks were highest at the start of the pandemic and started to wane over time:

The risk was changing all the time at [the start of the pandemic], and we did not really know… I think we are all a bit deadened to it now, probably, because it has been going on for quite a long time. (HP5_During)

Practitioners who were interviewed whilst recruitment was paused due to COVID-19 anticipated that it might be difficult to recruit and retain patients in the trial once it reopened due to patient and practitioner preferences for no further treatment:

My worry is that you’ll create a system where you open [the trial] up, everyone has got it open, but actually you’re not seeing people enrolling, because they’re worrying about maintenance. (HP4_During)

One practitioner explained that because of high levels of staff absence due to sickness or self-isolation at the height of the first wave, patients were no longer reviewed by a doctor after each cycle of induction. Although she found this challenging, she indicated that staff shortages had not been detrimental to patient care:

[Ordinarily,] we get [patients] reviewed by a doctor every [induction cycle]… So they do get … just a bit of extra TLC… [but] with COVID… the [specialist registrar] reviews that we did every cycle, got left just because we did not have the people around. But we would always ask [patients] if there were any problems... Just trying to mitigate the short staff levels was quite difficult. (HP5_During)

## Discussion

This is the first study that has compared patient and practitioner views and experiences of a clinical trial before and during COVID-19, although two other studies have explored patient and practitioner experiences of clinical research during the pandemic [36, 37]. Wyatt et al. reported on research staff experiences of the prioritisation of COVID-19 research when non-COVID-19 research was slowed or halted, patients attended fewer visits, and staff shifted towards working from home. Muwanguzi et al. reported patient and practitioner challenges of being involved in a trial of a work-place-based HIV self-testing kit during a time when many people were required to work from home. In contrast, the current study compared views and experiences before and during COVID-19 and also reported on patient and practitioner accounts of their interactions during these times.

In line with previous research [10], we found that before the pandemic, practitioner equipoise varied yet they described putting aside such biases to convey trial arms neutrally to patients. However, during the pandemic, practitioners reported limited evidence on which to inform risk/benefit discussions about randomisation and identified patient groups that they were less comfortable randomising to maintenance. Although research and guidance has been published to inform the treatment of patients with haematological cancers during the pandemic [38–40], it remains an ongoing management dilemma [38]. As emerging research suggests that patients undergoing maintenance to treat haematological cancers might not gain adequate protection from COVID-19 vaccines [20, 21], the dilemma regarding the risks and benefits of maintenance in the context of COVID-19 is unlikely to be resolved in the immediate future.

When comparing practitioners before and during COVID-19, we observed how COVID-19 influenced them in a way that was loaded against trial participation for CR patients. Practitioners were concerned maintenance might increase these patients’ risk of experiencing severe illness due to COVID-19, compared with the no further treatment arm. Consistent with other research on patients with haematological cancers [41], we found that trial participants were worried about contracting COVID-19 when attending hospital clinics during the first wave. Trial participants also reported reduced access to sources of trusted information and described uncertainty about their trial pathway due to more limited staff interactions.

The reported impact of COVID-19 on trial participant experiences and practitioner hesitancy to randomise some patients might be anticipated to lead to pronounced challenges in engaging patients in the trial [42]. However, while we interviewed one patient who withdrew from the trial during the pandemic, and other patients were continuing to review the decision to participate, we did not see pronounced changes in patient engagement with PETReA. This finding contrasts with a previous study that explored patient willingness to participate in a hypothetical trial during the pandemic [43]; the study found that around 1 in 5 cancer survivors anticipated they would be less likely to participate in a trial during COVID-19. During the pandemic, patients we interviewed, who had experienced this situation first-hand, were clear that they were keeping their participation in the trial under review, but they did not disengage. Rather they described discussions about trial arm risks and benefits with practitioners as more in-depth and collaborative, which they valued. Outside the pandemic, practitioners might feel reticent to discuss trials in-depth for fear of overwhelming patients, but at least at the height of the pandemic, patients found in-depth discussions helpful and this may have lessons that are transferable as the pandemic abates. Adopting communication strategies, such as having extended trial discussions and exploring patient treatment preferences (i.e. eliciting, acknowledging and balancing preferences) improves patient understanding and trial recruitment [44–46]. Table 3 provides a summary box of some of the key study findings and learning opportunities to enhance cancer trial communication and recruitment during uncertain times.

**Table 3:** Key study points and learning opportunities to enhance cancer trial communication and recruitment during uncertain times

• The pandemic introduced additional uncertainties in the context of PETReA, which altered practitioner and patient treatment preferences despite an absence of clear evidence about how COVID-19 influenced the risks of trial treatments.	
• Patients reported less face-to-face contact and fewer clinical appointments during COVID-19. They also described reduced access to sources of trusted information (e.g. research nurse contact) and uncertainty about the trial pathway following randomisation, compared to patients randomised before COVID-19.	
• Patients were worried about contracting COVID-19 when attending hospital clinics during the first wave. Patients’ risk perceptions of COVID-19 varied depending on the nature of their work, where they lived, who they lived with, and their behavioural strategies to avoid COVID-19 transmission.	
• We did not see pronounced changes in patient engagement with PETReA during COVID-19. Patients were clear that they were keeping their participation in the trial under review, but they did not disengage. The uncertainty surrounding COVID-19 led practitioners to emphasise that it was ok for patients to change their mind and withdraw from the trial, which helped patients continue with the trial during uncertain times.	
• Patients found the discussions with practitioners about the trial arm risks and benefits more in-depth and collaborative during COVID-19. Patients valued this and indicated that it supported them to continue with randomisation.	

In our study, patients were more likely to recall being told they were free to withdraw from the trial at any time during COVID-19, compared with before. Evidence from before COVID-19 indicates that a minority (21%) of audio-recorded trial consultations from a multi-centre UK-based trial included some discussion about patients being free to withdraw from the trial. Our findings, alongside this previous study, suggest that the uncertainty surrounding COVID-19 led practitioners to emphasise that it was ok for patients to change their minds and withdraw from the trial. Those interviewed during the pandemic also described being comforted in the knowledge that they could withdraw at any time, which helped them to continue with the trial. Current research indicates that, at least in written forms, an emphasis on the option to withdraw may diminish patient engagement with a trial [47], but the present findings indicate that a greater emphasis on this option during uncertain times helped patients to continue with the trial. Further research is needed to explore optimal ways to verbally communicate to patients that they are free to withdraw.

## Strengths and limitations

Our sample size is appropriate for a qualitative study designed for in-depth of case-oriented analysis that is vital to this method of investigation [48]. A major strength of this study was that we conducted interviews both before and during COVID-19. The original aim of the study was to identify opportunities to improve informed consent and recruitment to PETReA, but once the pandemic was announced, we adapted the topic guide and sought to compare patient and practitioner views and experiences before and during COVID-19. We sampled patients from NHS sites across the UK and our sample was diverse in terms of patient age, sex, socio-economic status, but we did not record ethnicity. We interviewed only one patient who declined PETReA (due to a high trial recruitment rate and few patients who declined being identified by sites); however, our sample varied in terms of response to induction (i.e. CR or PR) and trial arm allocation. We recruited patients from UK-based sites only, but anticipate that the results are more widely transferable as COVID-19 has impacted clinical trials globally [49–52] and many countries continue to have varying COVID-19 and vaccination rates. As highlighted in the results, the COVID-19 landscape continues to change, as do our risk perceptions and health-protective behaviours [53]. The interviews in our study that were conducted during COVID-19 took place between May and October 2020 and the experiences of patients and practitioners taking part in a clinical trial after this time may differ to those who were interviewed in this study. Irrespectively, the findings have implications that are relevant to optimising trial communication and conduct longer term.

## Conclusion

This qualitative interview study compared patient and practitioner views and experiences of a haematological cancer trial before and during COVID-19. We identified differences in views and experiences of the trial before and during COVID-19 and the findings indicated that changing views about the trial influenced trial communication with patients. Although practitioners became more hesitant about randomising some patients during the pandemic, the results suggest that consultations about randomisation became more in-depth and collaborative, which patients appreciated. Furthermore, the findings indicate that, compared with before the pandemic, during COVID-19 practitioners were more likely to emphasise to patients that they could withdraw from the trial. Our study suggests that these extended consultations can support patients to continue to engage in trials during uncertain times. The findings can be used to inform future trial communication training. It is especially relevant for trials recruiting patients who are clinically extremely vulnerable or who are at increased risk of poor COVID-19 outcomes despite vaccination.

## Supplementary Information

**Additional file 1.**

## Data Availability

Pseudo-anonymised data is available upon reasonable request via data custodian, Professor Bridget Young (byoung@liverpool.ac.uk)

## Glossary

PETReA (trial): Phase 3 evaluation of PET-guided, Response-Adapted therapy in patients with previously untreated, high tumour burden follicular lymphoma
Induction: Induction (or ‘first-line’) treatment delivered via 6–8 cycles over 4–6 months, which usually consists of chemotherapy and anti-CD20 antibody (typically ‘Rituximab’)
CT scan: A computerised tomography (CT) scan. In the context of PETReA, patients had a CT scan to assess anatomical response to induction.
PET-CT scan: Positron emission tomography (PET) image combined with computerised tomography (CT). In the context of PETReA, patients had a PET-CT scan to assess metabolic response to induction.
Complete metabolic response (CR): Complete metabolic response (CR) (or PET negative) response to induction. CR indicates that induction was highly effective, with no or minimal evidence of disease.
Partial response (PR): Partial response (PR) (or PET positive) response to induction. PR indicates that induction was effective, but shows that residual disease that requires treatment remains.
Maintenance: Maintenance is an antibody maintenance drug (anti-CD20 monoclonal treatment named Rituximab) delivered following induction as standard of care. One dose every 2 months, for 2 years.
Maintenance plus Lenalidomide: Maintenance (as above) delivered following induction, but alongside an additional drug, Lenalidomide. Lenalidomide is an immunomodulating drug. It is given orally for 3 in every 4 weeks, over two years.
No further treatment: No further treatment (unless relapse). Patient is monitored over 2 years.
