# Supplementary material for: Patient and health practitioner views and experiences of a cancer trial before and during COVID-19: qualitative study
Source: Trials. 2022 Jun 18;23:509. doi: 10.1186/s13063-022-06453-z (PMC9206129; doi:10.1186/s13063-022-06453-z)
Supplement: Supplementary file 1 — Additional file 1. [file 13063_2022_6453_MOESM1_ESM.pdf]

PETReA Information Study – Topic Guide for Healthcare Professionals

This provides an indication of the sorts of topics we plan to explore. We will always:

- Reiterate the aims and procedure of the interview before starting the interview
- Reassure the participant regarding confidentiality and check that the participant is happy to continue with interview before starting the interview.
- Tailor questions for each participant depending on their situation (e.g. whether they recruit patients into the study or not) and what is important for them as an individual.

As we interview more healthcare professionals over the course of the study and learn what questions are the most important to ask, we will refine or change questions.

<u>Views and experiences of the PETReA study</u>

1. What is your role in the PETReA study and how did you come about being involved in the study?

Prompts: Approximately how many patients have you spoken with about PETReA? Have you any previous experience of being part of a research study team?

2. What do you know about the PETReA study?

Prompts: How did it come about? Why is it being done? Are you aware of any evidence behind it? Do you think there's a need for the study? Why? Do you discuss the study with colleagues?

3. What information have you been given about the PETReA study?

Prompts: Discuss verbal and written information (including protocol/PIS). Did you receive too much/not enough information? Did you read the information provided? What did you think of it? Did it help/give you the information you needed? What suggestions would you have for improving it? Were there things you thought they had forgotten to include? Did you seek information from other sources (e.g. Internet, other people)? Explore which sources, what information that added or whether new questions were raised.

<u>Patient pathway</u>

4. Can you describe a typical patient pathway for a patient with follicular lymphoma?

Prompts: Diagnostic pathway? Symptoms? Duration? Who does the patient see? What is standard practice for a patient with follicular lymphoma? Who introduces the study? At which point in the patient pathway is it introduced? What do you think about the appropriateness of that? What are the eligibility criteria? What criteria are used to determine whether someone is PET negative/positive? What happens to borderline cases? Is clinical

judgement ever used? How is that explained to patients? What's the evidence for the cut off? How do patients respond to PET findings? Are there any cases where patients are eligible in theory but are not approached to participate? What happens to patients if they drop out of the study?

Main study discussion

5. Can you talk me through a recruitment discussion OR a conversation you've had with a patient about the PETReA study? Note: may not be applicable as some healthcare professionals do not recruit.

Acknowledge we have the recording but explain we're really interested in your point of view. How would you/recruiter introduce the study? Thinking back to the most recent patient, how did they respond to that initial introduction? How did that compare with other patients' reactions? Can you talk me through what happened next? Do you think they understood what was being said? Where there points that you needed to clarify or found difficult to explain? How were the PET findings explained? How was uncertainty about the best treatments explained? How was treatment escalation/de-escalation explained? How did you explain randomisation? How did you explain what happens if a patient declines the study? Drops out of the study? How do patients react to this information? What questions did they have? How do you respond if a patient states a preference? How did you feel about answering their questions? Did you feel you were able to take the time needed? What were your feelings about the conversation? What is the most important message for you to convey?

6. Can you talk me through a recruitment decision OR conversation about PETReA that did not go as smoothly as others/was less comfortable than others? Note: may not be applicable as some healthcare professionals do not recruit.

Prompts: What was said? How was it different? Did the patient have particular questions? How did you feel about answering their questions? What were your feelings about the conversation? More generally, have you noticed any situations or characteristics of patients that tend to make it harder to explain PETReA to a patient?

Views, understandings and experiences of the study and its arms

7. What do you think about treatment being stratified (that is, the interventions being dependent on the result of the PET scan)?

Prompt: How do you feel about this way of deciding what treatment a patient will receive?

8. What's your understanding of the treatments in the study?

Prompts: Feelings? Expectations? Worries? Perceptions of effectiveness of each treatment? Perceptions of side effects/risks of each treatment? Benefits? Long-term treatment plan for each? Follow-ups?

9. Do you think that PET negative patients would favour one of the interventions or not? How about PET positive patients?

Prompts: How would you respond? What do you see as the reasons for their preferences?

10. What are your thoughts about discontinuation of treatment for PET negative patients?

Prompts: Worries? Benefits? Feeling about this? Long-term outcome?

11. Are there any situations in which you would withdraw a patient from the study?

Prompts: Why? Do you think this is more likely to occur across a certain treatment arm?

<u>Views and experiences of randomisation</u>

12. What did you think about treatments being allocated by randomisation, i.e. by chance?

Prompts: How do you feel about this way of deciding what treatment patients get? Can you think of any better ways to allocate people to treatment arms?

<u>Follow-up</u>

13. What happens after patients finish treatment?

Prompts: Can you tell me a bit about follow-up? What happens after patients have finished treatment? How much follow-up contact do you have with patients? What kind of things do patients ask about after they finish treatment?

<u>Final thoughts</u>

14. What are your reasons for taking part in this Information Study?

Prompts: What would you like to see from this study? How would you like us to feed the findings of this study back?

15. Is there anything else you would like to tell me?

Prompts: About PETReA? Taking part in research in general? Are there any questions you would like to ask?

Thank you for taking the time to talk to me
